# Self-organized synchronization of digital phase-locked loops with delayed coupling in theory and experiment

**DOI:** 10.1371/journal.pone.0171590

**Published:** 2017-02-16

**Authors:** Lucas Wetzel, David J. Jörg, Alexandros Pollakis, Wolfgang Rave, Gerhard Fettweis, Frank Jülicher

**Affiliations:** 1 Max Planck Institute for the Physics of Complex Systems, Nöthnitzer Str. 38, 01187 Dresden, Germany; 2 Center for Advancing Electronics Dresden, cfaed, 01062 Dresden, Germany; 3 Vodafone Chair - Mobile Communications Systems, TU Dresden, 01062 Dresden, Germany; Lanzhou University of Technology, CHINA

## Abstract

Self-organized synchronization occurs in a variety of natural and technical systems but has so far only attracted limited attention as an engineering principle. In distributed electronic systems, such as antenna arrays and multi-core processors, a common time reference is key to coordinate signal transmission and processing. Here we show how the self-organized synchronization of mutually coupled digital phase-locked loops (DPLLs) can provide robust clocking in large-scale systems. We develop a nonlinear phase description of individual and coupled DPLLs that takes into account filter impulse responses and delayed signal transmission. Our phase model permits analytical expressions for the collective frequencies of synchronized states, the analysis of stability properties and the time scale of synchronization. In particular, we find that signal filtering introduces stability transitions that are not found in systems without filtering. To test our theoretical predictions, we designed and carried out experiments using networks of off-the-shelf DPLL integrated circuitry. We show that the phase model can quantitatively predict the existence, frequency, and stability of synchronized states. Our results demonstrate that mutually delay-coupled DPLLs can provide robust and self-organized synchronous clocking in electronic systems.

## Introduction

In spatially extended electronic systems that require coordination of a large number of components, it is a challenge to provide a synchronized time reference [1–3]. Such systems include, e.g., global positioning systems, servers on the internet, multi-core and multi-processor computer architectures, network-on-chips, and large antenna and sensor arrays [4–7]. A general issue for synchronization of oscillators are signal transmission delays caused by finite signal propagation speed [8–10]. A processor running at 1GHz has a period of 1ns, the time in which signals send at the speed of light can travel 30cm. This implies significant transmission delays at length scales in the centimetre range for such frequencies. In most cases, the time reference in electronic systems relies on hierarchical concepts, i.e., a high quality master-clock feeds forward a synchronization signal into a clock-tree, entraining all other slave-clocks [11]. In such systems, signal transmission delays have to be compensated, e.g., all connections in a clock-tree have to be of equal length [11, 12]. Such strategies become space and energy inefficient for large systems [13]. Moreover, hierarchical entrainment is vulnerable to fluctuations and failures, e.g., due to noise and cross-talk [11, 14]. Hence it is a challenge to achieve precise and robust synchronization of autonomous clocking units [15, 16].

Robust self-organized synchronization without hierarchical structures has been found in large collections of oscillators in nature. In biological systems, such as neuronal networks, coupled genetic oscillators, cardiac pacemaker cells, and in flashing fireflies, synchronization is attained in a self-organized way [17, 18]. Through mutual coupling of individual oscillatory units, these systems are able to synchronize robustly in highly noisy environments and in the presence of considerable communication delays [19–24]. For instance, in neuronal systems, axonal conduction delays are typically in the range of 1-100 of milliseconds [25], and therefore a considerable fraction of the typical neural oscillation frequencies between 1 and 100 Hz. This is achieved in the absence of an entraining master clock. It has been shown that such a decentralized synchronization strategy enables robust self-organization of synchronous states also in analog electronic networks, thereby providing a position-independent time reference [26]. Recently, we have developed a phase oscillator description which captures the dynamics of digital electronic oscillators [27]. We also showed that electronic networks provide excellent experimental conditions to study the nonlinear dynamics of mutually coupled oscillators [28–30] in a controlled environment with adjustable system parameters and transmission delays [27].

Here, we study synchronization dynamics in networks of delay-coupled digital phase-locked loops (DPLLs). We show theoretically that such networks operating on digital signals can be formulated in terms of a phase oscillator description and study the existence, stability and the frequencies of different frequency synchronized states, e.g., checkerboard, m-twist and in-phase synchronized states. In contrast to implicit expressions for the frequencies of the synchronized states in analog systems, we here provide closed analytical expressions for the frequencies of synchronized states in digital systems. These can be obtained explicitly due to the piecewise linear coupling function. Furthermore, we discuss the role of the characteristic integration time related to signal filtering within the DPLLs. This integration time leads to memory effects that generate inertia-like behavior. This can lead to transient waves that travel through the network, initiated by local perturbations [31]. Our theoretical results are supported by experiments performed on real networks of off-the-shelf DPLL integrated circuitry. These experiments include networks of up to 9 DPLLs with different coupling topologies, such as nearest-neighbour coupled networks with open or periodic boundary conditions. In Section 1, we develop a phase model of *N* mutually delay-coupled DPLLs. In Section 2 and Section 3, the existence and stability of different types of frequency-synchronized solutions for networks of mutually coupled DPLLs are investigated. In Section 4, we use our theory to illustrate the behavior of DPLL systems for specific examples. In Section 5, we present experimental data for systems of mutually coupled DPLLs with different network topologies and show that the measurements can be understood quantitatively using our theory. Finally, conclusions are drawn in Section 6.

## 1 Phase model for networks of DPLLs

**Fig 1:**
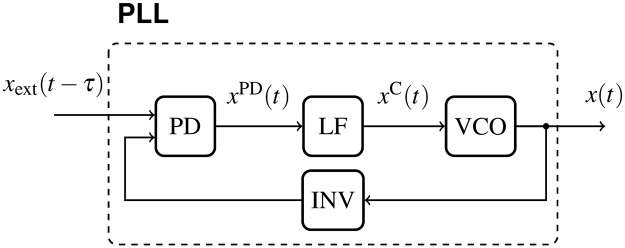
Signal flow model of the PLL considered in this paper. The delayed input signal to the PLL is denoted by *x*_ext_(*t* − *τ*) and its output signal by *x*(*t*). The output signal at the phase detector (PD) is denoted by *x*^PD^(*t*) and the output signal at the loop filter (LF) by *x*^C^(*t*). The output signal *x*(*t*) is generated by a voltage-controlled oscillator (VCO, square-wave symbol) and fed back to the PD. The feedback loop may contain a signal inverter (INV). © 2015 IEEE. Reprinted, with permission, from IEEE Proceedings: *Synchronization of mutually coupled digital PLLs in massive MIMO systems*.

The networks we propose consist of digital phase-locked loops (DPLL). These autonomous electronic oscillators allow inputs that can entrain their output signal by comparing the input signal to the feedback of the output signal [32]. Such clocks are widely used in electronics to control and synchronize components with respect to a master-clock signal. The essential building blocks of a DPLL are the phase detector (PD), the loop filter (LF) and the voltage-controlled oscillator (VCO). The PD can be implemented as an XOR gate and compares the external signal *x*_*l*_ to the feedback signal *x*_*k*_, see Fig 1. All external signals *x*_*l*_ and the feedback signal *x*_*k*_ are compared using XOR gates at the input for each input signal individually. High frequency components in the output signal $x_{k}^{\text{PD}}$ of the PD are filtered by the LF, a low pass filter allowing only low frequencies to pass. Then the output signal $x_{k}^{\text{C}}$ of the LF controls and modulates the VCO’s frequency. We consider a DPLL containing an optional signal inverter (INV) between the VCO and the PD (see 1). This inverter enables to control the properties of synchronization by introducing an additional phase shift *π*, independently of the instantaneous frequencies, as will be shown in Section 5. We first discuss a single DPLL to understand its function. In the following we extend our description to networks of arbitrarily coupled DPLL clocks.

### 1.1 Phase description of a single DPLL

We develop a phase description of a single DPLL receiving a time-delayed signal from a distant source. The components of the DPLL can be represented as follows. The VCO outputs a rectangular oscillation with amplitude 1,

$$\begin{array}{c} x\left(t\right)=\frac{1+\Pi (\phi (t))}{2}\;, \end{array}$$ (1)

where *ϕ*(*t*) is the phase and Π is the square-wave function with Fourier representation

$$\begin{array}{c} \Pi \left(\phi \right)=\frac{4}{\pi }\sum_{i=0}^{\infty }\frac{\sin([2i+1]\phi )}{2i+1}\;. \end{array}$$ (2)

**Fig 2:**
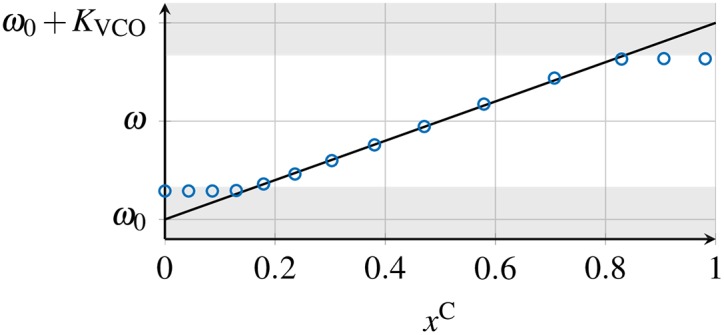
Frequency response of a VCO. Typical dynamic frequency response of a VCO to an external control voltage *x*^C^: linear approximation used in Eq (5) (solid curve), measured VCO response curve from the DPLLs in our experimental setup (circles). The shaded areas display the VCO clipping region, where the response saturates. © 2015 IEEE. Reprinted, with permission, from IEEE Proceedings: *Synchronization of mutually coupled digital PLLs in massive MIMO systems*.

Π is 2*π*-periodic and takes the value −1 for −*π* < *ϕ* < 0, the value 1 for 0 < *ϕ* < *π*, the value 0 for *ϕ* = 0 and *ϕ* = *π*. The PD compares the external input signal *x*_ext_ with the signal of the internal feedback *x* using an XOR operation. We account for transmission delays with a single delay time *τ* in the input signal,

$$\begin{array}{ccc} x^{\text{PD}}\left(t\right) & = & x_{\text{ext}}\left(t-\tau \right)⊕x\left(t\right)\\ & = & x_{\text{ext}}\left(t-\tau \right)\cdot \bar{x}\left(t\right)+\bar{x_{\text{ext}}}\left(t-\tau \right)\cdot x\left(t\right)\;, \end{array}$$ (3)

where ⊕ denotes the XOR operation and $\bar{x}\left(t\right)=1-x\left(t\right)$. The output signal *x*^PD^ of the PD is filtered by the LF whose output signal *x*^C^ is given by

$$\begin{array}{c} x^{\text{C}}\left(t\right)=\int_{0}^{\infty }\text{d}u\;p\left(u\right)\;x^{\text{PD}}\left(t-u\right)\;, \end{array}$$ (4)

where *p*(*u*) denotes the impulse response of the LF and satisfies $\int_{0}^{\infty }\text{d}u\;p\left(u\right)=1$. This control signal *x*^C^ is passed to the VCO and determines the VCO’s oscillation frequency according to

$$\begin{array}{c} \dot{\phi }\left(t\right)=\omega_{0}+K_{\text{VCO}}x^{\text{C}}\left(t\right)\;, \end{array}$$ (5)

where *ω*_0_ is the minimal frequency of the VCO for zero input and *K*_VCO_ is its sensitivity. We here consider a linear frequency response of the VCO, such that the frequency response is proportional to the control signal *x*^C^. Fig 2 shows this linear response together with the measured response curve from our experimental setup, discussed in Section 5. We consider the loop filter as an ideal low-pass filter that perfectly damps high frequency components, see A. Using Eqs (3) and (4) in Eq (5), we obtain

$$\begin{array}{c} \dot{\phi }\left(t\right)≃\omega +K\int_{0}^{\infty }\text{d}u\;p\left(u\right)\;\Delta (\phi_{\text{ext}}\left(t-\tau -u\right)-\phi \left(t-u\right)) \end{array}$$ (6)

where, for compactness of notation, we have defined *ω* = *ω*_0_ + *K*_VCO_/2 and *K* = *K*_VCO_/2 and where Δ is the triangle-wave function with Fourier representation

$$\begin{array}{c} \Delta \left(\phi \right)=-\frac{8}{\pi^{2}}\sum_{i=0}^{\infty }\frac{\cos([2i+1]\phi )}{{\left(2i+1\right)}^{2}}\;. \end{array}$$ (7)

Δ is piecewise linear with first derivative Δ′(*ϕ*) = −2/*π* for −*π* < *ϕ* < 0, slope Δ′(*ϕ*) = 2/*π* for 0 < *ϕ* < *π* and slope 0 for *ϕ* = 0 and *ϕ* = *π*. Moreover, Δ is 2*π*-periodic.

### 1.2 Networks of mutually coupled DPLLs

**Fig 3:**
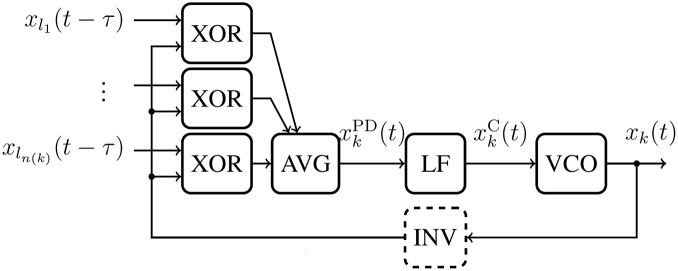
Signal flow of a DPLL with multiple delayed input signals. Each input signal *x*_*l*_*i*__ (*i* = 1, …, *n*(*k*)) undergoes the XOR operation with the feedback signal individually. The resulting signals are averaged (AVG). © 2015 IEEE. Reprinted, with permission, from IEEE Proceedings: *Synchronization of mutually coupled digital PLLs in massive MIMO systems*.

Networks of *N* mutually delay-coupled DPLLs can be analyzed using an extended version of the phase model Eq (6). If there is more than one input signal to a DPLL, each input is processed with the feedback by an XOR gate individually. Subsequently all signal paths are joined into an analogue average which yields the PD’s output signal *x*^PD^ (see Fig 3). The phase model for a network of *N* coupled identical DPLLs and equal transmission delays reads

$$\begin{array}{c} {\dot{\phi }}_{k}\left(t\right)≃\omega +\frac{K}{n(k)}\sum_{l=1}^{N}c_{kl}\int_{0}^{\infty }\text{d}u\;p\left(u\right)\Delta (\phi_{l}\left(t-\tau -u\right)-\phi_{k}\left(t-u\right))\;, \end{array}$$ (8)

where *ϕ*_*k*_ is the phase of oscillator *k* ∈ {1, …, *N*}. The connection topology between all DPLLs is described by the coupling matrix $C=(c_{kl})$ with *c*_*kl*_ ∈ {1, 0}, where *c*_*kl*_ = 1 indicates a connection between DPLL *k* and DPLL *l*. The coupling strength is normalized by the number of input signals, *n*(*k*) = ∑_*l*_ *c*_*kl*_, generating the average input signal to the LF of DPLL *k*. Eq (8) describe a network of *N* coupled DPLLs, taking explicitly into account a filter impulse response *p*(*u*) and identical transmission delays *τ*. Identical transmission delays can, e.g., be achieved in a regular square lattice with nearest-neighbor coupling.

In large systems of delay-coupled oscillators, many different dynamic states can exist, e.g., phase-synchronized and frequency-synchronized states as well as more complex phase patterns such as spirals, traveling waves, and irregular or chaotic states [33, 34]. Here, we are interested in synchronized states with a constant collective frequency and constant phase relations between oscillators. In the following two sections, we analyze the existence and stability of two important classes of such states, the in-phase synchronized states (Sec. 2) and the *m*-twist synchronized states (Sec. 3).

## 2 In-phase synchronized states

### 2.1 Frequency of the in-phase synchronized state

**Fig 4:**
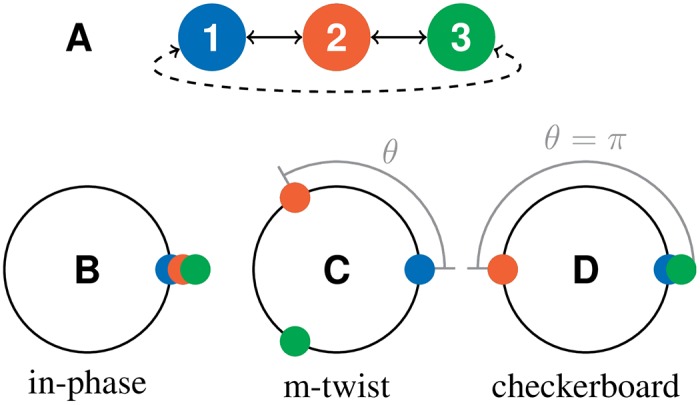
*m*-twist synchronized states. (A) Coupling topologies for a system of 3 mutually coupled DPLLs. (B–D) Synchronized states with constant collective frequency and constant phase relation between the oscillators for a system of three mutually coupled oscillators with periodic (cases B,C) or open boundary conditions (cases B,D): (B) in-phase synchronized state, (C) m-twist synchronized state and (D) checkerboard synchronized state.

In globally in-phase synchronized states (see Fig 4), all DPLLs evolve with the same collective frequency *Ω* and have no phase lag relative to each other,

$$\begin{array}{c} \phi_{k}\left(t\right)=\Omega t\;. \end{array}$$ (9)

Inserting Eq (9) in Eq (8), we find a condition for the collective frequency,

$$\begin{array}{c} \Omega =\omega +K\Delta (\Omega \tau )\;. \end{array}$$ (10)

**Fig 5:**
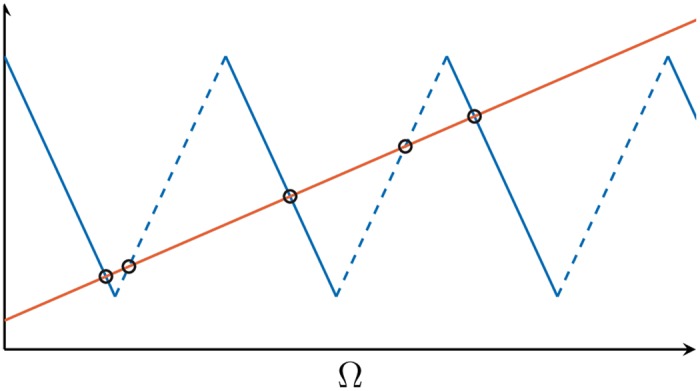
Global frequencies Ω for triangular coupling functions. Graphical representation of the lhs (red) and rhs (blue) of Eq (10) as a function of Ω. In this example, we find 5 coexisting solutions for the chosen *τ*-value, indicated by circles.

Here we have used $\int_{0}^{\infty }\text{d}u\;p\left(u\right)=1$, *n*(*k*) = ∑_*l*_ *c*_*kl*_, and the fact that Δ is even. An in-phase synchronized state exists if Eq (10) has a solution in Ω. Explicit solutions to Eq (10) can be obtained graphically as the intersection of the lhs and the rhs of Eq (10), see Fig 5. The intersection points can be expressed explicitly as

$$\begin{array}{c} \Omega_{j}^{\pm }=\frac{\omega -(1\pm 4j)K}{1\mp 2K\tau /\pi }\;, \end{array}$$ (11)

where the sign ± refers to intersections with the rising and falling slopes of Δ, respectively. The indices *j* fall within a range j∈N between *A* and *B* − 1/2 for $\Omega_{j}^{+}$ and between *A* and *B* + 1/2 for $\Omega_{j}^{-}$, where *A* = (*ω* − *K*)*τ*/2*π* and *B* = (*ω* + *K*)*τ*/2*π*. For a given set of parameters, multiple solutions with different collective frequencies can exist. The collective frequency is independent of the number *N* of oscillators and the coupling matrix C. The number of solutions depends on the parameters, in particular on the transmission delay *τ*. In the following section, we analyze the stability of these states.

### 2.2 Stability of the in-phase synchronized state

In-phase synchronized states are robust against fluctuations if small perturbations decay. Performing a linear stability analysis, we obtain the stability properties of the state given by Eq (9) [35, 36]. We consider the dynamics of small perturbations *q*_*k*_(*t*), defined by

$$\begin{array}{c} \phi_{k}\left(t\right)=\Omega t+q_{k}\left(t\right)\;. \end{array}$$ (12)

The linear dynamics of the perturbation is obtained by expanding Eq (8) to first order

$$\begin{array}{c} {\dot{q}}_{k}\left(t\right)=\frac{\alpha }{n(k)}\sum_{l=1}^{N}c_{kl}\int_{0}^{\infty }\text{d}u\;p\left(u\right)[q_{l}\left(t-\tau -u\right)-q_{k}\left(t-u\right)], \end{array}$$ (13)

with respect to *q*_*k*_, where *α* = *KΔ*′(−*Ωτ*). Using the definition of the triangle-wave function Δ, Eq (7), *α* explicitly reads

$$\begin{array}{c} \alpha =\frac{2K}{\pi }\Pi \left(-\Omega \tau \right)\;. \end{array}$$ (14)

where Π is the square-wave function Eq (2). Using the exponential ansatz *q*_*k*_(*t*) = *v*_*k*_*e*^*λt*^ in Eq (13), with complex frequency *λ* and perturbation *v*_*k*_, we obtain the characteristic equation

$$\begin{array}{c} \lambda v_{k}=\alpha \hat{p}\left(\lambda \right)\sum_{l=1}^{N}d_{kl}(v_{l}e^{-\lambda \tau }-v_{k}) \end{array}$$ (15)

where the Laplace transform of the impulse response, $\hat{p}\left(\lambda \right)=\int_{0}^{\infty }\text{d}u\;{\text{e}}^{-\lambda u}p\left(u\right)$, is the transfer function of the LF and *d*_*kl*_ = *c*_*kl*_/*n*(*k*) are the components of the normalized coupling matrix $D=(d_{kl})$. We solve Eq (15) for the unknowns *λ* and **v** = (*v*_1_, …, *v*_*N*_). To this end, Eq (15) can be rewritten in the form ρ(λ)v=Dv, where $\rho \left(\lambda \right)={\text{e}}^{\lambda \tau }\left(\lambda \hat{p}{\left(\lambda \right)}^{-1}\alpha^{-1}+1\right)$. This reveals that **v** must be an eigenvector of the matrix D, which satisfies Dv=ζv, and where *ζ* is the corresponding eigenvalue. Hence, *λ* must satisfy *ρ*(*λ*) = *ζ*, which explicitly reads

$$\begin{array}{c} \frac{\lambda }{\hat{p}\left(\lambda \right)}+\alpha \left(1-\zeta e^{-\lambda \tau }\right)=0\;. \end{array}$$ (16)

Note that each value of *ζ* generates an infinite discrete set Λ_*ζ*_ of solutions *λ*. If D is diagonalizable, arbitrary perturbation vectors can be expressed as linear combinations of the eigenvectors **v**. The matrix D always has the special eigenvector **v** = (1, …, 1) with *ζ* = 1, corresponding to a global phase shift of all oscillators. This eigenvector exists because ∑_*l*_ *d*_*kl*_ = 1 for all *k* by definition of D. Since a global phase shift does not perturb the synchrony of the system, we do not consider the corresponding perturbation modes in the following. The long-time evolution of the perturbation is dominated by

$$\begin{array}{c} \lambda_{0}=\sigma +\text{i}\beta \;, \end{array}$$ (17)

which is the solution with the largest real part within the set of solutions ⋃_*ζ* ≠ 1_Λ_ζ_ [26]. The real part *σ* of *λ*_0_ is the perturbation response rate. The in-phase synchronized state is stable if and only if *σ* < 0. The imaginary part *β* is a frequency that leads to a periodic modulation of the phase. The perturbation *q*_*k*_(*t*) thus generates side-bands in the oscillator signal with frequencies Ω ± *β* [26].

## 3 *m*-twist and checkerboard synchronized states

### 3.1 Frequency of *m*-twist and checkerboard synchronized states

We now consider frequency-synchronized states with a fixed phase difference between neighboring DPLLs (see Fig 4). In one dimension, the phase profile of such *m*-twist states has the form

$$\begin{array}{c} \phi_{k}\left(t\right)=\Omega t+k\theta_{m}\;. \end{array}$$ (18)

Introducing periodic boundary conditions enforces that the phase difference can only take the values

$$\begin{array}{c} \theta_{m}=\frac{2\pi m}{N}\;, \end{array}$$ (19)

where *m* ∈ {0, …, *N* − 1} is the total number of 2*π*-phase increments along the ring. The case *m* = 0 corresponds to the in-phase synchronized state described in Sec. 2. For *N* even, the case *m* = *N*/2 corresponds to a checkerboard pattern, i.e., a phase difference of *θ*_*N*/2_ = *π* between neighboring oscillators. The in-phase and checkerboard synchronized states also exist in the case of open boundary conditions.

Inserting the Ansatz Eq (18) into Eq (8) using the nearest-neighbor coupling matrix in one dimension *c*_*kl*_ = *δ*_*k*,*l*−1_ + *δ*_*k*,*l*+1_ + *δ*_*k*,*N*_*δ*_*l*,1_ + *δ*_*k*,1_*δ*_*l*,*N*_, we find the condition

$$\begin{array}{c} \Omega =\omega +\frac{K}{2}[\Delta \left(\Omega \tau +\theta_{m}\right)+\Delta \left(\Omega \tau -\theta_{m}\right)] \end{array}$$ (20)

for the collective frequency, which depends on *m*. Here we have used $\int_{0}^{\infty }\text{d}u\;p\left(u\right)=1$, *n*(*k*) = 2, and the fact that Δ(*x*) = Δ(−*x*). For each Ω that solves Eq (20), an *m*-twist synchronized state exists. The collective frequency Ω depends on the number *N* of oscillators through the phase difference *θ*_*m*_. Depending on the value of the transmission delay *τ*, Eq (20) can have multiple solutions representing different *m*-twist synchronized states with the same phase difference *θ*_*m*_ but different collective frequencies. A closed form solution analogous to Eq (11) for the in-phase synchronized state for Eq (20) is given in B.

### 3.2 Stability of the *m*-twist synchronized state

We study the linear stability of the states given by Eq (18) by using the Ansatz

$$\begin{array}{c} \phi_{k}\left(t\right)=\Omega t+k\theta_{m}+q_{k}\left(t\right)\;, \end{array}$$ (21)

where *q*_*k*_ is a small perturbation to the *m*-twist synchronized state. The linearized dynamics of the perturbations *q*_*k*_ can be written as

$$\begin{array}{c} {\dot{q}}_{k}\left(t\right)=\sum_{\pm }\frac{\alpha_{\pm }}{2}\int_{0}^{\infty }\text{d}u\;p\left(u\right)[q_{k\pm 1}\left(t-\tau -u\right)-q_{k}\left(t-u\right)] \end{array}$$ (22)

where the sum runs over + and −, with *α*_±_ = *K*Δ′(−*Ωτ* ± *θ*_*m*_). Using the definition of the triangle-wave function Δ, Eq (7), *α*_±_ explicitly read

$$\begin{array}{c} \alpha_{\pm }=\frac{2K}{\pi }\Pi \left(-\Omega \tau \pm \theta_{m}\right)\;. \end{array}$$ (23)

Inserting the exponential ansatz *q*_*k*_(*t*) = *v*_*k*_*e*^*λt*^ into Eq (22), the characteristic equation reads

$$\begin{array}{c} \lambda v_{k}=\frac{\hat{p}\left(\lambda \right)}{2}\sum_{\pm }\alpha_{\pm }(v_{k\pm 1}{\text{e}}^{-\lambda \tau }-v_{k})\;, \end{array}$$ (24)

where we imply periodic boundary conditions. As in Sec. 2.2, this equation can be rearranged and written in vector notation as γ(λ)v=Ev, where $\gamma \left(\lambda \right)={\text{e}}^{\lambda \tau }\left(2\lambda \hat{p}{\left(\lambda \right)}^{-1}+\alpha_{+}+\alpha_{-}\right)$ and the matrix E has entries *e*_*kl*_ = *α*_ + _*δ*_*k*,*l*−1_+*α*_−_*δ*_*k*,*l*+1_ + *α*_+_*δ*_*k*,*N*_*δ*_*l*,1_ + *α*_−_*δ*_*k*,1_*δ*_*l*,*N*_. Hence, for this equation to possess solutions in *λ*, *γ*(*λ*) must be an eigenvalue of E. The eigenvalues *η*_*j*_ of E are given by

$$\begin{array}{c} \eta_{j}=\left(\alpha_{+}+\alpha_{-}\right)\cos(\frac{2\pi j}{N})+\text{i}\left(\alpha_{+}-\alpha_{-}\right)\sin(\frac{2\pi j}{N})\;, \end{array}$$ (25)

where *j* = 0, …, *N* − 1. Note that the matrix E and hence the eigenvalues *η*_*j*_ depend on both the coupling matrix C and the other parameters through *α*_+_ and *α*_−_. The characteristic equation *γ*(*λ*) = *η* with *η* being an eigenvalue of E can be written as

$$\begin{array}{c} \frac{\lambda }{\hat{p}\left(\lambda \right)}+\frac{\alpha_{+}+\alpha_{-}-\eta {\text{e}}^{-\lambda \tau }}{2}=0\;. \end{array}$$ (26)

Eq (26) has a discrete infinite set of solutions for each *η*.

### 3.3 Stability of the checkerboard synchronized state

So far, we have considered periodic boundary conditions. However, the checkerboard synchronized state, characterized by a phase difference *θ*_*m*_ = *π* between neighboring oscillators, also exists for open boundary conditions. The characteristic equation which determines the linear stability of this state is

$$\begin{array}{c} \frac{\lambda }{\hat{p}\left(\lambda \right)}+\alpha_{+}\left(1-\eta {\text{e}}^{-\lambda \tau }\right)=0\;, \end{array}$$ (27)

where *α*_+_ has been defined in Eq (23) and the eigenvalues *η*_*j*_ are given by

$$\begin{array}{c} \eta_{j}=\cos(\frac{j\pi }{N-1})\;, \end{array}$$ (28)

using the fact that *α*_+_ = *α*_−_ for *θ* = *π*.

## 4 Specific DPLL systems as examples

We now discuss the collective frequency and the stability of the in-phase, 1-twist, and checkerboard synchronized states of three coupled DPLLs. We consider both a ring and a chain coupling topology (see Fig 4).

### 4.1 Collective frequency

**Fig 6:**
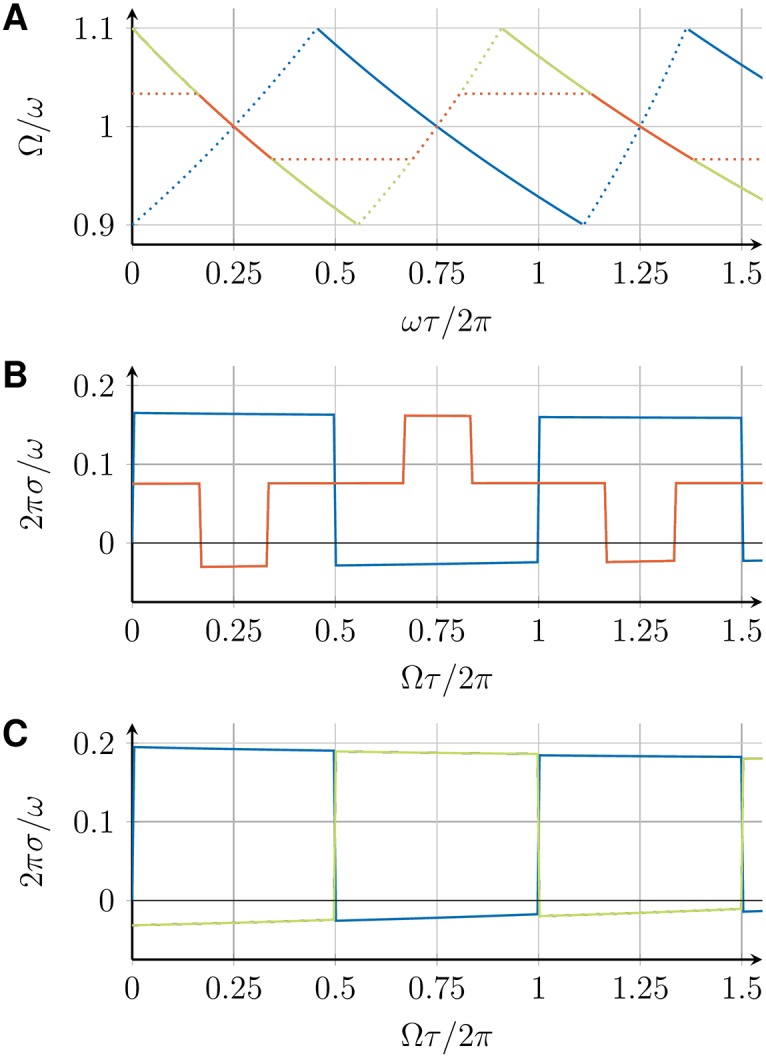
Global frequencies Ω and perturbation response rates *σ* for three coupled DPLLs. (A) Collective frequency Ω as a function of the transmission delay *τ* for the in-phase (blue), *m*-twist with *m* = 1, 2 (red), and checkerboard (green) synchronized states, as determined from Eqs (10) and (20). Solid lines indicate stable solutions, dotted lines indicate unstable solutions. (B) Perturbation response rate *σ*, Eq (17), as a function of Ω*τ* for different synchronized states in a ring of 3 coupled DPLLs (color code as in panel A). (C) Perturbation response rate *σ* as a function of Ω*τ* for different synchronized states in a chain of 3 coupled DPLLs (color code as in panel A). All results are shown for a system of identical PLLs with $\omega =2\pi \;\text{rad}\;\;{\text{s}}^{-1}$, *K* = 0.1*ω*, $\hat{p}$ given by Eq (29) with *a* = 1 and *ω*_*c*_ = 0.01*ω*.

The collective frequency Ω for the in-phase, 1-twist, and checkerboard state, determined by Eqs (10) and (20), depends on the intrinsic frequency *ω* of the VCOs, the coupling strength *K*, and the transmission delay *τ*. Fig 6A shows Ω for all three states as a function of *τ*. For a fixed value of *τ*, multiple states with different collective frequencies can be stable simultaneously. The dependence of the collective frequency on the transmission delay is qualitatively different for the in-phase and the 1-twist synchronized states. The range of possible collective frequencies is larger for the in-phase state.

### 4.2 Stability and perturbation response rates

For *σ* < 0, the synchronized state is linearly stable and the perturbation response rate −*σ* is a measure of the time scale of synchronization. This time-scale or equivalently the perturbation response time can be calculated as *t*_*c*_ = −1/*σ*. It corresponds to the time in which perturbations have decayed to *q*_*k*_(*t*_*c*_)/*q*_*k*_(0) = 1/*e* and can be obtained using *q*_*k*_(*t*) = *q*_*k*_(0)*e*^*λt*^. Fig 6 shows *σ* as a function of Ω*τ* for the in-phase and 1-twist synchronized states in a ring of 3 coupled DPLLs (see Fig 6B) and for the in-phase and the checkerboard synchronized states in a chain of 3 coupled DPLLs (see Fig 6C). Note that there are parameter regions for which none of these states is linearly stable, indicating that the system attains more complex dynamical states, whose characterization is outside the scope of the present work. For the special case of no delay, *τ* = 0, we find *σ* = 0 for all synchronized states. Hence, these states are marginally stable and small perturbations persist.

**Fig 7:**
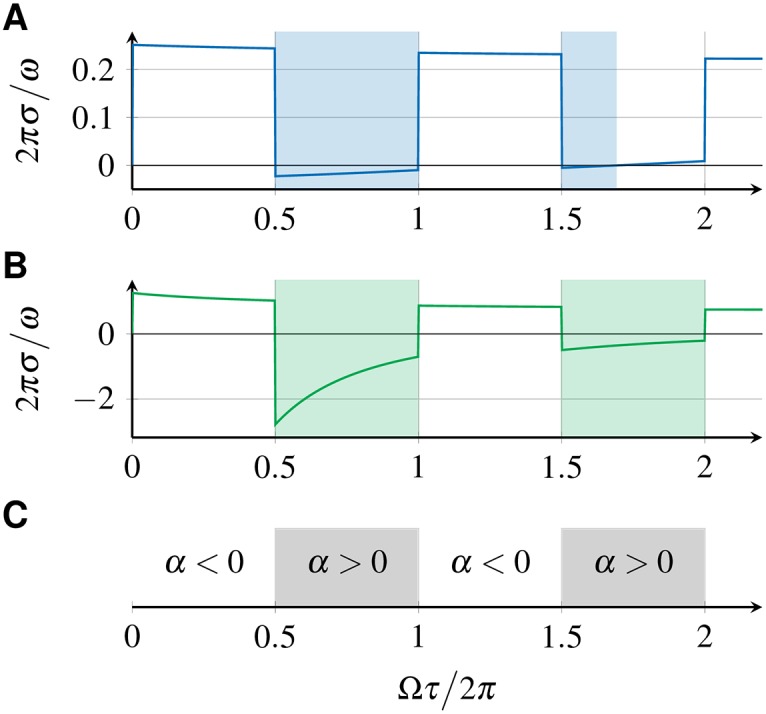
Perturbation response rate *σ* of the in-phase synchronized state for two coupled DPLLs. Perturbation response rate *σ* of the in-phase synchronized state in a system of two mutually coupled DPLLs, defined by Eq (17), for $\hat{p}$ given by Eq (29) with (A) *a* = 1 and *ω*_*c*_ = 0.01*ω* and (B) *a* = 0. (C) Sign of *α*, Eq (14), as a function of the transmission delay. In all plots, shaded regions indicate where the in-phase synchronized state is stable. Regions where *α* > 0 coincide with the stability regions in (B). The other parameters are $\omega =2\pi \;\text{rad}\;\;{\text{s}}^{-1}$, *K* = 0.1*ω*.

The values of the transmission delay for which synchronized states are stable depend on the transfer function $\hat{p}$ of the loop filter via Eqs (16) and (26). Here we consider the large class of loop filters with the transfer function

$$\begin{array}{c} \hat{p}\left(\lambda \right)=\frac{1}{{\left(1+\frac{\lambda }{a\omega_{\text{c}}}\right)}^{a}}\;, \end{array}$$ (29)

where *a* is the order of the filter and *ω*_c_ is the cutoff frequency [37]. Fig 7 shows the perturbation response rate *σ* for two examples in which the filter transfer function $\hat{p}$ is given by Eq (29) with filter order *a* = 1 and *a* = 0, where the latter case corresponds to no filtering. Shaded regions indicate stable in-phase synchronized solutions. Note that the regions where the in-phase synchronized state is stable differs for these two cases. It has been shown earlier that for the case of no filtering (*a* = 0), the in-phase synchronized state is stable if and only if *α* > 0 with *α* given by Eq (14) [38]. Fig 7 thus shows that in the presence of a filter, linear stability is no longer determined by the sign of *α*. Hence, the presence of a filter can change the stability of synchronized states. Moreover, filtering also affects the time scales of synchronization as has been shown earlier [26]. Therefore, effects of filters have to be taken into account in models of coupled PLLs in order to obtain correct stability properties. For filters of first order (*a* = 1), the effect of the filtering can be understood as inertia of the phase variable due to characteristic integration time of the filter, see C.

## 5 Experimental measurements on coupled DPLLs

**Fig 8:**
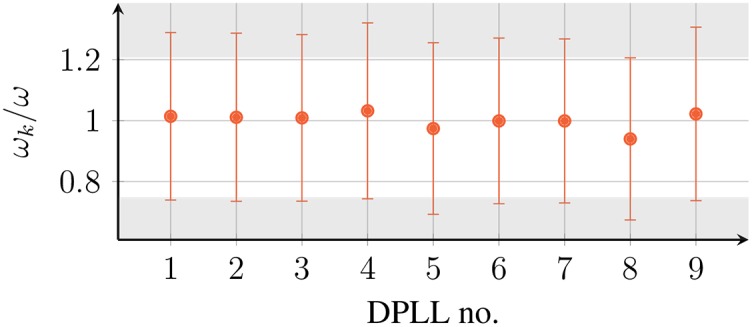
Dynamic frequency range of all 9 DPLLs specified in Table 1. Red dots indicate the intrinsic frequency *ω*, bars indicate the frequency range where the VCO response is linear (see Fig 2). Shaded areas indicate the system’s clipping regions; outside these regions the response of all VCOs is linear.

**Fig 9:**
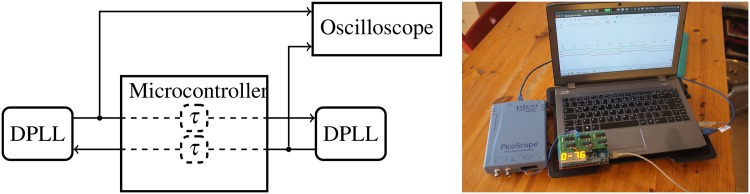
Experimental setup, sketch and photograph. Two DPLLs coupled through a microcontroller, which artificially introduces a transmission delay. An oscilloscope samples the periodic signals of the DPLLs. © 2015 IEEE. Reprinted, with permission, from IEEE Proceedings: *Synchronization of mutually coupled digital PLLs in massive MIMO systems*.

We designed prototypes to conduct experiments with networks of mutually coupled DPLLs to compare the synchronization properties of a real system to our theoretical predictions. The prototype networks were set up with DPLLs (CD4046B [39], specifications given in Table 1 and Fig 8), a Digilent ChipKit Max32 microcontroller [40], and a PicoScope 2205 Mixed-Signal oscilloscope [41] (see Fig 9). We choose the kHz regime for our prototypes to make sampling of the signals simple, and because the effects introduced by the delay only depend on the relation between delay and period of the uncoupled oscillators. If signals can be transmitted with about two thirds of the speed of light [9], clocks operating in the kHz range would require connection lengths of the order of kilometers to achieve significant transmission delays. The necessary transmission delays were achieved by artificially holding back the signals in a microcontroller according to a chosen value of the delay. We measure the output signals of the DPLLs using an oscilloscope. From the data the phase time series of each of the DPLLs of the network was obtained. We use this data to measure the exponential relaxation time to a synchronized state and frequency of the synchronized state. The measurement parameters are given in Table 2.

**Table 1:** Specification of the DPLLs in the experimental setup.

DPLL no.	*ω*/2*π*	*K*^VCO^/2*π*	*ω*_c_/2*π*	*a*
1	1011	813	14 Hz	1
2	1008	816	14 Hz	1
3	1006	809	14 Hz	1
4	1029	845	14 Hz	1
5	971	833	14 Hz	1
6	996	804	14 Hz	1
7	996	796	14 Hz	1
8	937	787	14 Hz	1
9	1019	842	14 Hz	1
Mean	997	816	14 Hz	1
Standard deviation	2.8%	2.5%		

**Table 2:** Measurement parameters of the experiments.

Description	Value
Signal amplitudes	5 V
Sampling interval	10 *μ*s
Number of samples	109224

We performed measurements on three different systems, analyzing for each system different synchronized states: (A.) a system of 2 mutually coupled DPLLs, (B.) a ring of 3 mutually coupled DPLLs, and (C.) a two-dimensional square lattice of 3 × 3 mutually coupled DPLLs with periodic and open boundary conditions. Note that system (B.) corresponds to one of the examples discussed in the last section. The specifications of the used DPLLs are given in Table 1. After a system is started, we first let the DPLLs oscillate independently during a randomly chosen time and then turn on the coupling. Because of small differences in intrinsic frequencies, oscillators have an arbitrary phase difference at the moment they are coupled. Subsequently, the system settles into a synchronized state for a wide range of delay times. All experimental results are averages over up to 40 independent measurements with the standard deviation being smaller than the symbol size in all plots presented. The number of data points that are averaged depends on how likely the respective synchronized state is attained starting from arbitrary initial conditions. We compare these measurements to results of our phase model using the average DPLL parameters for the intrinsic frequency *ω* and the coupling strength *K*.

### 5.1 Two mutually coupled DPLLs

**Fig 10:**
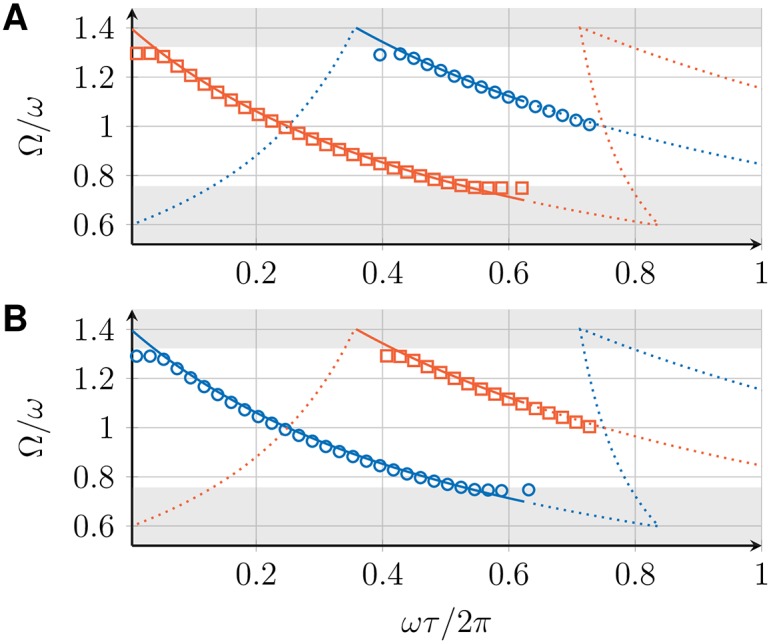
Experimental measurements of the collective frequencies Ω for two coupled DPLLs. Collective frequency Ω of the in-phase (blue circles) and checkerboard synchronized state (red squares) for two mutually coupled DPLLs as a function of the transmission delay *τ*. Symbols show experimental data points. Lines show analytical results of the phase model, Eq (10), where solid blue lines denote in-phase solutions and red lines denote checkerboard solutions. The shaded areas display the VCO clipping region. (A) Operation mode with signal inverter deactivated. (B) Operation mode with signal inverter activated. © 2015 IEEE. Reprinted, with permission, from IEEE Proceedings: *Synchronization of mutually coupled digital PLLs in massive MIMO systems*.

We first discuss the simplest case of two mutually coupled DPLLs (no. 1 and no. 2 in Table 1). Two types of synchronized states are observed: the in-phase and the checkerboard synchronized state (see Fig 4). Fig 10 shows the measured collective frequency Ω for different values of the transmission delay, together with the results obtained from the phase model, Eq (10). Which of the two synchronized states is found depends on the value of the transmission delay. For certain ranges of the delay *τ* both synchronized states coexist and the system is bistable. In the coexistence region, synchronized states are selected stochastically after transient dynamics. The VCO response becomes nonlinear and saturates in the VCO clipping region (see Fig 2). Outside these VCO clipping regions, where the VCO response is linear, the measured values of the collective frequency are in good agreement with the results of the phase model, see Fig 8. Inside the VCO clipping regions, the linear response approximation of the VCO’s dynamic frequency, Eq (5), becomes inaccurate and the measured collective frequency deviates from the theoretical results. Adding a signal inverter in the feedback loop (see Fig 1) exchanges the collective frequency and the stability of the in-phase and the checkerboard synchronized state (compare Fig 10A and 10B). Alternatively, the inverter can also be added to the output of each PLL or each input with the same result.

**Fig 11:**
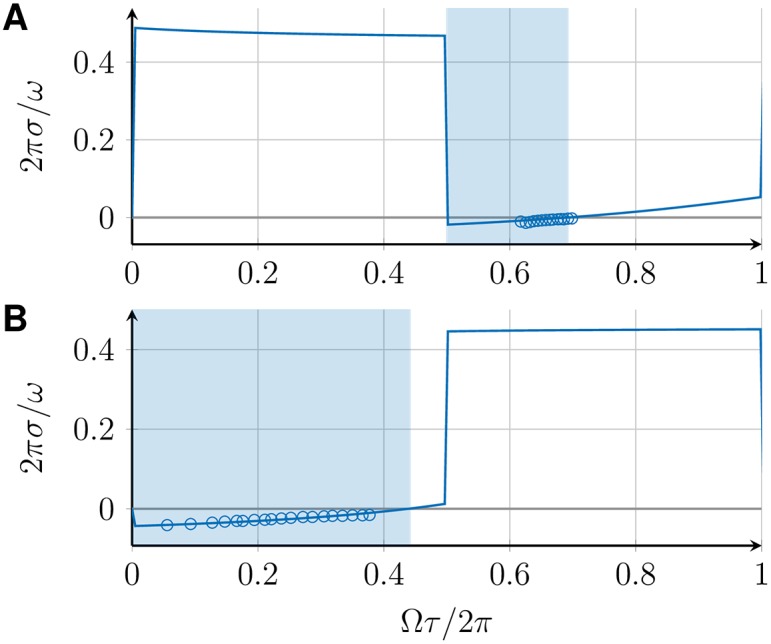
Experimental measurements of the perturbation response rate *σ* for two coupled DPLLs. Perturbation response rate *σ*, Eq (17), of the in-phase synchronized state for a system of two mutually coupled DPLLs for (A) deactivated inverter and (B) activated inverter. Symbols show experimental data points. Lines show results of the phase model, solutions to Eq (16) with *ζ* = −1, obtained numerically using a Levenberg- Marquardt algorithm [42, 43] with initial value *λ*_0_ = 0.1 + *i* 0.1. The shaded areas display the stability regions of the in-phase synchronized state as given by the phase model. © 2015 IEEE. Reprinted, with permission, from IEEE Proceedings: *Synchronization of mutually coupled digital PLLs in massive MIMO systems*.

From experimental measurements, we obtained the exponential relaxation time *σ*^−1^ of the phase difference between the two DPLLs by fitting an exponential function to the data. Experimental data were obtained outside the VCO clipping regions and for values of the delay for which the in-phase synchronized state is stable (*σ* < 0). Fig 11 shows the experimental and theoretical results for the perturbation response rate *σ*. Again, the experimental results are in quantitative agreement with the results from the phase model.

### 5.2 Ring of three mutually coupled DPLLs

**Fig 12:**
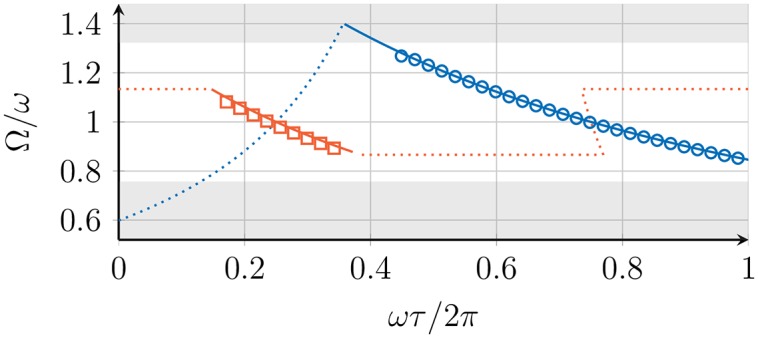
Experimental measurements of the collective frequencies Ω for three coupled DPLLs. Collective frequency Ω of the in-phase (blue circles) and *m*-twist synchronized states with *m* = 1, 2 (red squares) for a ring of 3 mutually coupled DPLLs as a function of the transmission delay *τ*. Symbols show experimental data points. Color code as in Fig 10.

We now study a ring of three mutually coupled DPLLs (no. 1, 2, and 3 in Table 1). In this experimental setup, we observe in-phase, 1-twist, and equivalent 2-twist synchronized states. Fig 12 shows the measured collective frequency Ω of the in-phase (0-twist) and the 1-twist state for different values of the transmission delay, together with the results from the phase model, Eq (20). The behavior of these solutions is in quantitative agreement with the results of the phase model.

### 5.3 Square lattice of 3 × 3 mutually coupled DPLLs

**Fig 13:**
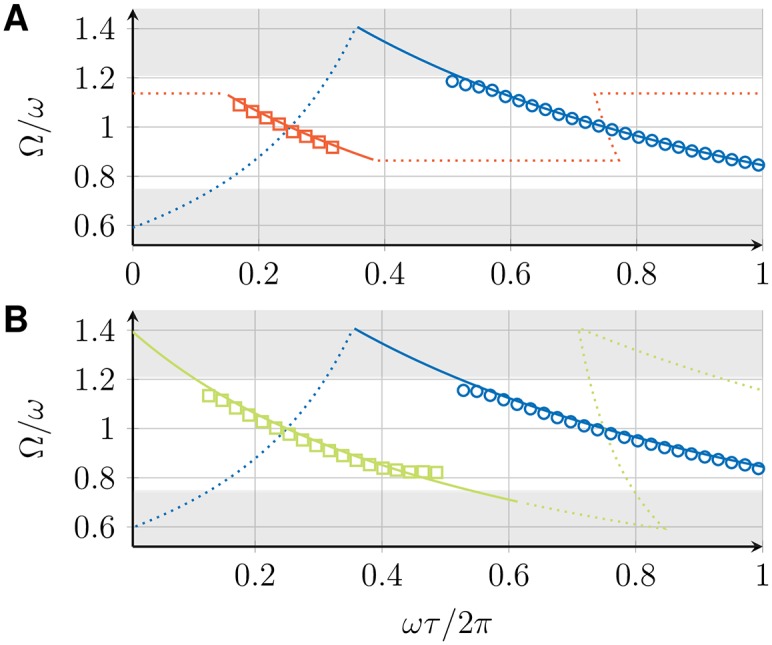
Experimental measurements of the collective frequencies Ω for nine coupled DPLLs. Collective frequency Ω of different synchronized states for a square lattice of 3 × 3 mutually coupled DPLLs as a function of the transmission delay *τ*. (A) In-phase synchronized state (blue circles) and all combinations of *m*_1_-*m*_2_-twist synchronized states with *m*_1_, *m*_2_ ≠ 0 (red squares) for a system with periodic boundary conditions. (B) In-phase synchronized state (blue circles) and checkerboard synchronized state (green squares) for a system with open boundary conditions. The shaded areas display the system’s clipping region as shown in Fig 8. Color code as in Fig 10.

We now study a square lattice of 3 × 3 mutually coupled DPLLs with periodic and open boundary conditions (no. 1–9 in Table 1). Depending on the type of boundary condition, we observe in-phase, *m*-twist, or checkerboard synchronized states. For a nearest-neighbor coupled system in two dimensions, *m*-twists can occur in each dimension individually, corresponding to constant phase offsets *θ*_*m*_1__ and *θ*_*m*_2__ in *x* and *y*-direction, respectively. For the special case of *θ*_*m*_1__ = *θ*_*m*_2__, the results for the collective frequency Ω are identical to those for a one-dimensional ring discussed in Sec. 3. Similarly, the checkerboard synchronized state in two dimensions has the same collective frequency as the checkerboard state in one dimension. Fig 13 shows the measured collective frequency Ω of the in-phase, *m*-twist, and checkerboard synchronized states of a 3 × 3 DPLL array for different values of the transmission delay, together with the results of the phase model, Eq (20). Fig 13A shows the in-phase and *m*-twist states for periodic boundary conditions, Fig 13B shows the in-phase and checkerboard states for open boundary conditions. The behavior of these solutions is in quantitative agreement with the results of the phase model.

## 6 Conclusion and discussion

In this paper, we have shown in theory and in experiments that global synchronization can be achieved by mutually coupling DPLLs with transmission delays. We used a phase oscillator description for networks of digital PLLs, taking into account the effects of filtering and transmission delays, to analyze frequency-synchronized states. The collective frequencies of in-phase, *m*-twist, and checkerboard synchronized states were obtained analytically. We analyzed the stability of those states and showed, how the DPLL’s filters and transmission delays in the network affect synchronization, in particular the collective frequency and the time scales of synchronization. Specifically, the collective frequency of a synchronized state in general differs from the intrinsic frequency of the DPLLs. In the presence of transmission delays the collective frequency of a synchronized state deviates from the intrinsic frequencies of the DPLLs. We found that it is essential to take into account the dynamic properties of filtering as it has profound effects on the stability properties. In particular, filtering introduces new stability transitions compared to delay-coupled phase oscillators without filters, which were discussed in [38]. Filtering leads to an inert system behavior. This can most easily be seen for the case of filters of first order, *a* = 1, in which the dynamic equations can be expressed by a second-order differential equation comprising an inertial term, see C. For spatially extended systems of coupled phase oscillators with inertia, it has been shown that perturbations can initiate transient waves that travel through the system and that might affect the synchronization behavior [31]. Our approach of mutually coupled distributed DPLLs enables frequency-synchronized states for many oscillators and thus has the potential to scale up to large systems. It is particularly relevant for, e.g., massive MIMO systems due to considerable delays induced by typical spacing between antennas. However, it can also be applied to other systems where a large number of clocks has to be synchronized.

We designed experiments to test the predictions of our phase model in real systems of coupled DPLLs. Our measurements of the collective frequency and the synchronization relaxation times show that our theory can quantitatively describe the behavior of mutually delay-coupled DPLL systems. All dynamic states discussed here in theory have been shown to exist in real networks of coupled DPLL systems in our experiments. Centralized state-of-the-art systems require a master clock together with complicated clock trees to ensure that the entrained DPLLs receive the clock signal at the same time. In contrast, the system proposed here relies on a regular lattice geometry and is therefore readily implemented and trivially scalable. Moreover, wiring distances between components are kept to a minimum. Through systematically taking into account the effects of transmission delays that enable synchronization, these delays become a design parameter rather than a limitation of the system.

The theory presented here is a practical tool to design systems of coupled DPLLs for technical applications and to predict their behaviors. We showed that with XOR phase detectors, the stability of such synchronized states depends strongly on the transmission delays in the coupling and the properties of the loop filter. Other theoretical studies for networks of mutually-coupled PLLs with flip-flop phase detectors report that the effects of time-delays can be neglected [44]. We have considered an idealized system of delay-coupled DPLLs without phase noise. Phase noise is present in any real system of coupled DPLLs and can lead to fluctuations such as cycle slips. While our experiments, in which phase noise is inevitable, are nevertheless well described by our theory, a detailed study of the effects of phase noise is currently conducted in our group. The problem of efficiently booting such systems into synchrony and the role of phase noise are interesting topics for future research.

## A Approximation of the control signal

Here we determine the frequency contributions of the PD’s output signal $x_{k}^{PD}\left(t\right)$ and discuss the impact of the low pass filter, i.e. the damping, on the these components. The Fourier representation of the square wave function Π and the triangle function Δ are given by Eqs (2) and (7). Hence, evaluating Eq (3) yields,

$$\begin{array}{ccc} x_{k}^{\text{PD}} & = & \frac{1}{2}-\frac{8}{\pi^{2}}\sum_{ij}\frac{\sin\left(a_{i}\phi_{l,\tau }\right)\sin\left(a_{j}\phi_{k}\right)}{a_{i}a_{j}}\\ & = & \frac{1}{2}-\frac{4}{\pi^{2}}\sum_{i}\frac{\cos(a_{i}\left(\phi_{l,\tau }-\phi_{k}\right))}{a_{i}^{2}}\\ & & -\frac{4}{\pi^{2}}\sum_{i\neq j}\frac{\cos(a_{i}\phi_{l,\tau }-a_{j}\phi_{k})}{a_{i}a_{j}}\\ & & -\frac{4}{\pi^{2}}\sum_{ij}\frac{\cos(a_{i}\phi_{l,\tau }+a_{j}\phi_{k})}{a_{i}a_{j}}\;, \end{array}$$ (30)

where *a*_*i*_ = 2*i* + 1 and *ϕ*_*l*,*τ*_(*t*) = *ϕ*_*l*_(*t* − *τ*). The different sums in the second identity represent different frequency components. From Eq (5), it can be seen that ${\dot{\phi }}_{k}-{\dot{\phi }}_{l}=O\left(K_{\text{VCO}}\right)$. Hence, the first sum in the second identity contains low frequency components. The lowest frequency components in the second sum are given by the terms with *i* + *j* = 1. From Eq (5), it can be seen that $a_{i}{\dot{\phi }}_{k}-a_{1-i}{\dot{\phi }}_{l}=O\left(2\omega_{0}\right)$ for *i* ∈ {0, 1} and hence, the second sum contains only high frequency components. The lowest frequency component in the third sum is given by the term with *i* = *j* = 0. From Eq (5), it can be seen that ${\dot{\phi }}_{k}+{\dot{\phi }}_{l}=O\left(2\omega_{0}\right)$ and hence, the third sum contains only high frequency components. Thus, using Eq (7), we can write $x_{k}^{\text{PD}}$ as

$$\begin{array}{c} x_{k}^{\text{PD}}\left(t\right)=\frac{1}{2}+\frac{1}{2}\Delta (\phi_{l}\left(t-\tau \right)-\phi_{k}\left(t\right))+R_{\text{HF}}\left(t\right)\;, \end{array}$$ (31)

where *R*_HF_ denotes the high frequency components of the signal. In Eq (6), we approximate $\int_{0}^{\infty }\text{d}u\;p\left(u\right)\;R_{\text{HF}}\left(t-u\right)≃0$, corresponding to ideal suppression of the high frequency components by the loop filter [32].

## B Collective frequency of the m-twist synchronized state

We state the explicit solutions to Eq (20) for the collective frequency Ω of the *m*-twist synchronized states. We use the piecewise linear behavior of the rhs of Eq (20) to obtain these solutions as intersection points between the lhs and rhs of Eq (20), analogously to the solutions for the in-phase synchronized state given in Section 2.1.

We define the constants *μ* and *ν* by

$$\begin{array}{ccc} \mu & = & \left\{ \begin{array}{cc} 0 & \theta_{m}\leq \pi \\ 1 & \theta_{m}>\pi \end{array}\;,\right.\\ \nu & = & \left\{ \begin{array}{cc} -1 & \pi /2\leq \theta_{m}\leq 3\pi /2\\ +1 & \text{else} \end{array}\;.\right. \end{array}$$ (32)

Potential intersection points between the lhs and the rhs of Eq (20) are given by

$$\begin{array}{c} \Omega_{*}^{\pm }=\omega \pm K\Delta \left(\theta_{m}\right) \end{array}$$ (33)

and

$$\begin{array}{c} \Omega_{j}^{\pm }=\frac{\omega -(1\pm 4j)\nu K}{1\mp 2\nu K\tau /\pi }\;. \end{array}$$ (34)

with j∈N. Whether these expressions provide actual intersection points and hence solutions for the collective frequency can be determined as follows. We define the condition Q(a,b;ψ) to be fulfilled if and only if

$$\begin{array}{c} \min(a,b)\leq [(\psi +\pi )\;\text{mod}\;2\pi ]-\pi <\max(a,b) \end{array}$$ (35)

and the condition P(a,b;ψ) to be fulfilled if Q(a,b;ψ) or Q(-a,-b;ψ) holds.

$\Omega_{*}^{+}$ is a solution if and only if the condition

$$\begin{array}{c} P(-\theta_{m}+\kappa \pi ,\theta_{m}-\kappa \pi ;\Omega_{*}^{+}\tau ) \end{array}$$ (36)

with *κ* = *μ*(1 + *ν*) + (1 − *ν*)/2 holds.

$\Omega_{*}^{-}$ is a solution if and only if the condition

$$\begin{array}{c} P(-\pi ,\nu \left[1-2\mu \right]\theta_{m}+[2\mu \nu -\frac{1+\nu }{2}]\pi ;\Omega_{*}^{-}\tau ) \end{array}$$ (37)

holds.

$\Omega_{j}^{\pm }$ is a solution if *j* falls within a range j∈N between *A*_*ν*_ and *B*_*ν*_ − 1/2 for $\Omega_{j}^{+}$ and between *A*_*ν*_ and *B*_*ν*_ + 1/2 for $\Omega_{j}^{-}$, where *A*_*ν*_ = (*ω* − *νK*)*τ*/2*π* and *B*_*ν*_ = (*ω* + *νK*)*τ*/2*π* and, in addition, the condition

$$\begin{array}{c} P(\theta_{m}-\pi ,2\mu \pi -\theta_{m};\Omega_{j}^{\pm }\tau ) \end{array}$$ (38)

holds.

## C First order signal filtering in a PLL and inertia

Models of coupled oscillators with inertia are an active and emerging research field [45–48]. Here we show that for a LF of first order, the filter integral in the phase model Eq (8) can be rewritten and yields a second order delay-differential equation including inertia. The phase model for first order filters with *a* = 1, given in Eq (8), and finite phase history starting at *t* = 0 can be expressed in Laplace space as

$$\begin{array}{c} \left(1+\lambda b\right)\lambda {\hat{\phi }}_{k}\left(\lambda \right)-\left(1+\lambda b\right)\phi_{k}\left(0\right)=\frac{\omega }{\lambda }+\omega b+K{\hat{x}}_{k}^{PD}\left(\lambda \right)\;, \end{array}$$ (39)

where ${\hat{\phi }}_{k}\left(\lambda \right)$ denotes the Laplace transform of the phase and ${\hat{x}}_{k}^{PD}\left(\lambda \right)$ the Laplace transform of the phase detector signal. Transformation back into time domain yields

$$\begin{array}{c} b{\ddot{\phi }}_{k}\left(t\right)+{\dot{\phi }}_{k}\left(t\right)=\omega +\left[\omega -{\dot{\phi }}_{k}\left(0\right)\right]b\delta \left(t-0\right)+Kx_{k}^{PD}\left(t\right)\;, \end{array}$$ (40)

where $x_{k}^{PD}\left(t\right)=n_{k}^{-1}\sum_{l}d_{kl}h\left[\phi_{l}\left(t-\tau \right)-\phi_{k}\left(t\right)\right]$. The term $[\omega -{\dot{\phi }}_{k}\left(0\right)]b\delta \left(t-0\right)$ denotes a phase kick at *t* = 0 with *b*, the characteristic integration time of the filter, which shifts the phases of all oscillators determined by the initial conditions. Comparison with Eq (8) reveals that the signal filtering with a filter of first order can be rewritten as an inertia term of the phase variable.

## Supporting information

S1 File Experimental data file. S1_File.zip.
(ZIP)
